# PyZebrascope: An Open-Source Platform for Brain-Wide Neural Activity Imaging in Zebrafish

**DOI:** 10.3389/fcell.2022.875044

**Published:** 2022-05-19

**Authors:** Rani Barbara, Madhu Nagathihalli Kantharaju, Ravid Haruvi, Kyle Harrington, Takashi Kawashima

**Affiliations:** ^1^ Department of Brain Sciences, Weizmann Institute of Science, Rehovot, Israel; ^2^ Max-Delbrück-Centrum for Molecular Medicine in the Helmholtz Association, Berlin, Germany; ^3^ Humboldt University of Berlin, Berlin, Germany; ^4^ Oak Ridge National Laboratory, Oak Ridge, TN, United States

**Keywords:** zebrafish, lightsheet microscopy, open source, software, image processing

## Abstract

Understanding how neurons interact across the brain to control animal behaviors is one of the central goals in neuroscience. Recent developments in fluorescent microscopy and genetically-encoded calcium indicators led to the establishment of whole-brain imaging methods in zebrafish, which record neural activity across a brain-wide volume with single-cell resolution. Pioneering studies of whole-brain imaging used custom light-sheet microscopes, and their operation relied on commercially developed and maintained software not available globally. Hence it has been challenging to disseminate and develop the technology in the research community. Here, we present PyZebrascope, an open-source Python platform designed for neural activity imaging in zebrafish using light-sheet microscopy. PyZebrascope has intuitive user interfaces and supports essential features for whole-brain imaging, such as two orthogonal excitation beams and eye damage prevention. Its camera module can handle image data throughput of up to 800 MB/s from camera acquisition to file writing while maintaining stable CPU and memory usage. Its modular architecture allows the inclusion of advanced algorithms for microscope control and image processing. As a proof of concept, we implemented a novel automatic algorithm for maximizing the image resolution in the brain by precisely aligning the excitation beams to the image focal plane. PyZebrascope enables whole-brain neural activity imaging in fish behaving in a virtual reality environment. Thus, PyZebrascope will help disseminate and develop light-sheet microscopy techniques in the neuroscience community and advance our understanding of whole-brain neural dynamics during animal behaviors.

## Introduction

Animal behaviors occur through the collective actions of neurons distributed across the brain. Understanding such distributed neural dynamics in their entirety has been one of the central goals in neuroscience (Ahrens and Engert, 2015). Toward this goal, optical recording of neural activity at a brain-wide scale has become possible based on recent developments in genetically-encoded calcium indicators (Chen et al., 2013; Inoue et al., 2019) and volumetric fluorescence microscopy (Keller et al., 2008; Sofroniew et al., 2016; Voleti et al., 2019; Demas et al., 2021). Whole-brain neural activity imaging at cellular resolution was first demonstrated in larval zebrafish (Ahrens et al., 2013; Panier et al., 2013; Freeman et al., 2014) among other vertebrate model organisms using digital scanned laser light-sheet microscopy (DSLM) (Keller et al., 2008). DSLM excites sample fluorescence in multiple voxels along the light cone of the excitation beam, and rapid scanning of the excitation beam enables fast volumetric scans with high spatial resolution and low light toxicity. These advantages of DSLM are best exploited in the optically transparent brain of larval zebrafish. DSLM enabled studies of whole-brain neural dynamics during visually-evoked swimmming (Vladimirov et al., 2014; Chen et al., 2018), motor learning (Kawashima et al., 2016; Markov et al., 2021), learned helplessness (Mu et al., 2019), threat escape (Mancienne, 2021), and body posture change (Migault et al., 2018). It also revealed spontaneous noise dynamics across the brain (Ponce-Alvarez et al., 2018) and how those dynamics change during neural perturbations (Vladimirov et al., 2018; Yang, 2018) or administration of psychoactive reagents (Burgstaller et al., 2019). Further, it enabled high-speed voltage imaging on a single axial plane for recording membrane potential and spiking activity from a neural population during swimming in the midbrain (Abdelfattah et al., 2019) and the spinal cord (Böhm et al., 2022). These pioneering studies in zebrafish using DSLM expanded our understanding of diverse functionalities of the vertebrate brain.

**FIGURE 1:**
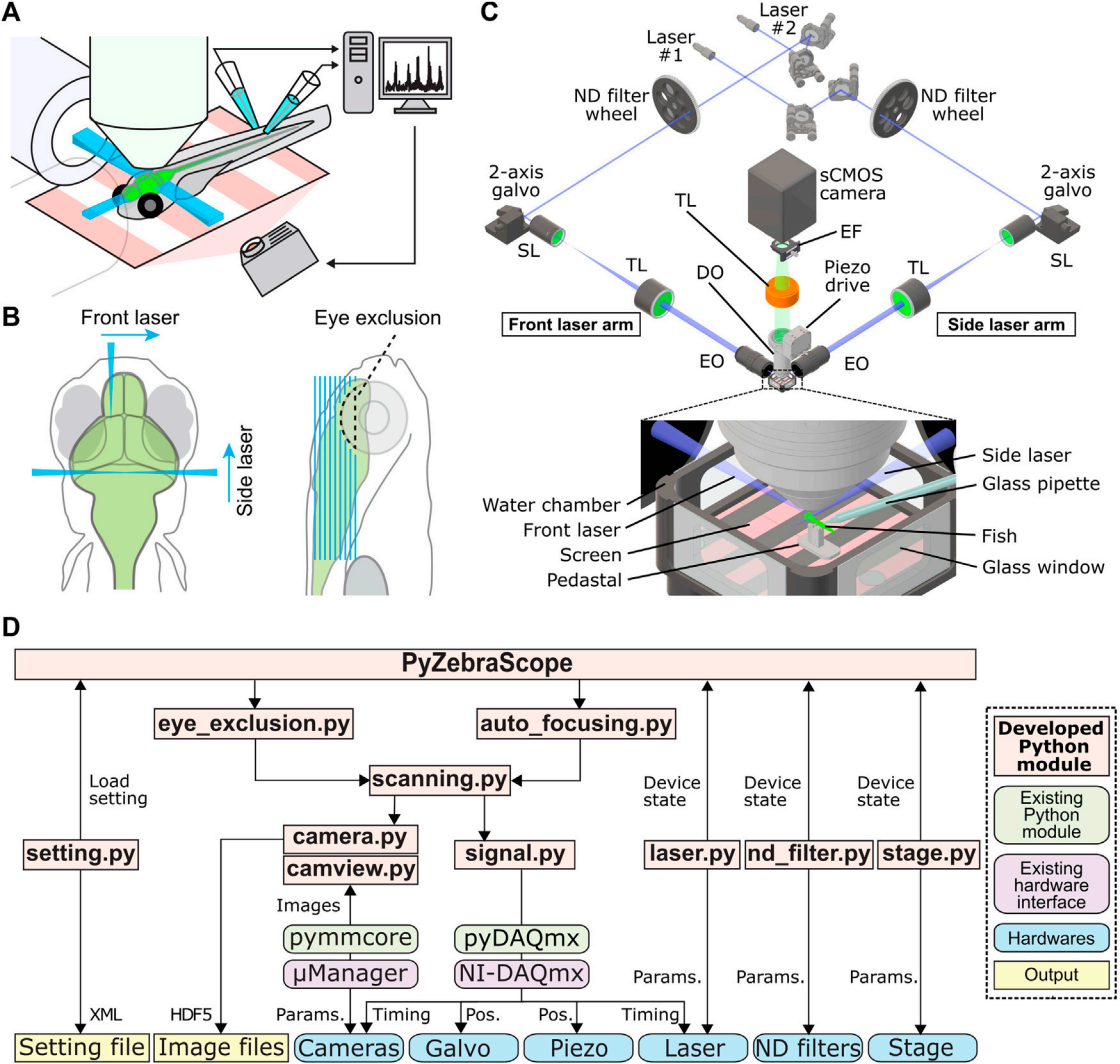
Our microscope setup and modular architecture of PyZebrascope for whole-brain neural activity imaging in zebrafish. **(A)** Schematic drawing of setups for whole-brain neural activity imaging in behaving zebrafish. Excitation objectives on the lateral and front sides of the fish illuminate excitation beams into the brain. The detection objective above the fish moves in the axial direction in sync with the motion of the excitation beams during the volumetric scan. We paralyze the muscle of the fish and record swim-related electrical signals from axonal bundles of spinal motoneurons by using two pipettes attached to the tail. The recorded signals are analyzed online and reflected in the motion of visual scenes projected below the fish. **(B)** Two-beam scanning during whole-brain imaging. The front laser beam scans areas between the eyes, and the side laser beam scans areas behind and above the eye. The side laser beam avoids eye damage by turning off when it overlaps with the eye. **(C)** Schematic of our light-sheet microscope and water chamber for imaging zebrafish. Detailed descriptions are in the main text. TL, tube lens; SL, scan lens; EO, excitation objective; DO, detection objective; EF, emission filter. **(D)** The architecture of PyZebrascope software. Our developed modules (beige), existing Python libraries (green), hardware drivers (purple), hardware (blue) and output files (yellow) are shown. Arrows show information flow between modules with labels such as “Pos.” (linear position information for devices) and “Params.” (device parameters).

Despite the above advantages of DSLM in achieving both high optical resolution and imaging speed compared to other large-scale volumetric imaging methods, such as structured illumination microscopy (Marques et al., 2020), multiphoton microscopy (Ahrens et al., 2012; Naumann et al., 2016; dal Maschio et al., 2017; Bruzzone, 2021), and light-field microscopy (Demas et al., 2021; Cong et al., 2017), it is still challenging to build a light-sheet microscope customized for zebrafish imaging and operate it for multiple experiments every day. These challenges come from the complexity of the microscope itself and its built-in parameters that the experimenter needs to manipulate. For example, light-sheet microscopes for whole-brain imaging in zebrafish (Figure 1A) typically consist of two excitation optical paths from the lateral and front sides of the fish and one optical detection path above the fish (Vladimirov et al., 2014; Migault et al., 2018; Markov et al., 2021; Mancienne, 2021) (Figure 1B). The fish is fixed in a water chamber and needs to be precisely maneuvered into focal points of the excitation and detection objectives. Moreover, it is necessary to prevent laser illumination into the eyes to secure fish’s vision for behavioral tasks and prevent eye damage (Figure 1B). This configuration requires the experimenters to set at least ∼20 parameters (camera exposure time per plane, number of planes per volume, the start and end positions for 2d motion for each excitation beam, light intensity and on/off timing for lasers, the start and end positions for detection objective motions, 3-dimensional positions for the fish chamber, and parameters for eye exclusion). The first studies (Ahrens et al., 2013; Vladimirov et al., 2014) on whole-brain imaging in zebrafish were made possible by using software custom-developed and maintained by a commercial entity, which charges high-priced service costs and does not provide service globally. This situation prevented the dissemination of the technology in a flexible and customizable manner. Past progress of optical microscopy in neuroscience has been driven by open-source software (Pologruto et al., 2003; Voigt et al., 2022) for microscope control written in a programming language widely used in academics, such as ScanImage (Pologruto et al., 2003) for multiphoton microscopy. Hence, developing an open-source platform for light-sheet microscopy dedicated to neural activity imaging in zebrafish is necessary.

Recently, software interface for light-sheet microscopy in zebrafish, μSPIM (Saska et al., 2021), was developed based on the open-source microscope control platform μManager (Edelstein et al., 2014) in Java/C programming language. Although this study only demonstrated partial volumetric imaging of the zebrafish brain based on single excitation beam scanning, it opened the possibility of developing an advanced open-source platform for light-sheet microscopy at a larger scale. Such software will reduce the laboriousness of imaging experiments by providing control of numerous device parameters on a simple and intuitive interface. Its implementation in a standard programming language for researchers will facilitate the addition of innovative functionalities toward their research goals by importing modules from a broad developer ecosystem for hardware control, signal processing, computer vision, and machine learning.

Toward this goal, we developed PyZebrascope, an open-source Python platform designed for whole-brain imaging in zebrafish using light-sheet microscopy. PyZebrascope supports essential features for whole-brain imaging, such as two orthogonal excitation beams and eye damage prevention. Its user interfaces allow the users to adjust parameters for lasers, filters, beam scanning, camera, sample positions, and eye damage prevention intuitively. Its multi-threaded camera module can handle stable high data throughput from multiple cameras. It is also possible to add advanced algorithms for microscope control and image processing developed in the Python community (Van Der Walt et al., 2014; Harris et al., 2020). As a proof of concept, we implemented a novel GPU-based automatic algorithm for maximizing the image resolution in the brain by precisely aligning the excitation beams to the image focal plane, which is usually a time-consuming and indecisive process for the experimenter. Lastly, we demonstrated that PyZebrascope enables whole-brain imaging in zebrafish behaving in a virtual reality environment. Thus, PyZebrascope is a versatile platform for disseminating and advancing technology for large-scale neural activity imaging in zebrafish and will accelerate our understanding of whole-brain neural dynamics during animal behaviors.

## Methods

### Software and Hardware

PyZebrascope: All the modules of PyZebrascope were written in Python programming language on Spyder IDE (https://www.spyder-ide.org/). We used a PC for microscope control that has Windows 10 operation system, two processors (Intel Xeon Gold 6,244), 384 GB of DDR4 memory, a GPU (nVidia GeForce GTX1660), and a 15 TB solid-state drive (Micron 9300 Pro, >3 GB/s writing/reading speed). We followed manufacturers’ manuals for connecting devices, including the camera, data acquisition board and sample stages.

We benchmarked the resource usage and disk writing speed of PyZebrascope (Figure 3) by using psutil package (https://github.com/giampaolo/psutil) in Python. We identified the process id of PyZebrascope by using Windows task manager and monitored its CPU and memory usage at an interval of 5 s in a separate Python instance while PyZebrascope records camera images for 30 min. Disk writing speed was also monitored using psutil by tracking the increase of disk usage in the above solid-state drive at the same time interval.

We calculated the image resolution measure in Figure 4B as half the wavelength of the identified maximum frequency after Fourier and polar transformations based with the below formula

$$r_{image}=\frac{\left[image\;dimension\right]*\left[pixel\;dimension\right]}{2*F}$$

whereas [pixel dimension] is 0.405 μm per camera pixel in our setup and *F* is in the identified maximum frequency component (pixel distance after the polar transformation).

Light-sheet microscope: We designed a custom light-sheet microscope that has a virtual reality setup for behavioral recordings, two optical paths for excitation beams, two cameras for fluorescence detection, and additional space for future implementation of the third optical path for excitation beam and neural perturbations (Supplementary Figure S1). We designed this microscope using Inventor Professionals software (Autodesk), and our CAD model is available upon request. We listed commercially available parts and custom-designed parts for our light-sheet microscope in Supplementary Table S1 and their overall costs in Supplementary Table S2.

### Zebrafish Experiments

We used a 6-day old transgenic zebrafish that pan-neuronally express nuclear-localized, genetically-encoded calcium indicators (Dana et al., 2019) and co-express RFP in tph2+ neurons (Tg(HuC:H2B-GCaMP7f)^jf96^ and Tg(tph2:epNTR-TagRFP)^jf41^, courtesy of Dr. Misha Ahrens) for the imaging experiment. The zebrafish was immobilized and mounted to an imaging chamber as described previously (Kawashima et al., 2016). Briefly, the fish larvae were immobilized by bath application of α-Bungarotoxin (B1601, Fisher Scientific, 1 mg/ml) dissolved in external solution (in mM: 134 NaCl, 2.9 KCl, 2.1 CaCl2, 1.2 MgCl2, 10 HEPES, 10 glucose; pH 7.8; 290 mOsm) for 25–30 s and embedded in agarose on a custom-made pedestal inside a glass-walled chamber with a diffusive screen underneath the fish (Figure 1C). Agarose around the head was removed with a microsurgical knife (#10318-14, Fine Science Tools) to minimize the scattering of the excitation laser. Laser power from the side beam path was, on average, approximately 21 µW. The distance between the fish and the display was about 4 mm.

We performed a fictive recording of fish’s swim patterns during a motor learning task (Kawashima et al., 2016). Electric signals from motor neuron axons in the tail were recorded using borosilicate pipettes (TW150-3, World Precision Instruments) pulled by a horizontal puller (P-1000, Sutter) and fire-polished by a microforge (MF-900, Narishige). The pipettes were filled with fish-rearing water and connected to the tail using minimal negative pressure. Swim signals were recorded using an amplifier (RHD2132 amplifier connected to RHD-2000 interface board, Intan Technologies). We used custom-written Python software (available upon request) for executing the same algorithms for closed-loop virtual reality experiments as the software used in previous studies (Kawashima et al., 2016; Abdelfattah et al., 2019). It samples signals from the above amplifier at 6 kilohertz, automatically detects swim events, and moves the visual stimulus projected below the fish in a closed-loop with a delay of 35 milliseconds. The motor learning task consisted of 12 repetitions of a 157-s session, where we presented different types of tasks (7-s training, 15-s training, and 30-s training) in a pseudo-random manner.

Data analysis: We processed acquired imaging data on a Linux server in High Performance Computing (HPC) division in the Weizmann Institute of Science. This server has two Xeon processors (Xeon Gold 6,248, Intel), 384 GB RAM, 13-TB SSD array, and a GPU computing board (Tesla V100, nVidia). We performed data processing using custom Python scripts that execute the same algorithms as those established in our previous work (Kawashima et al., 2016). All the analyses of imaging data were performed on a remote JupyterLab environment (https://jupyterlab.readthedocs.io/).

Briefly, we first registered time-series images from the same Z-planes by using phase correlation algorithms on the above GPU. We then examined residual drifts in the lateral and axial directions and discarded data with excessive drifts (>5 μm) in either direction. We then identified individual neurons that express nuclear-localized GCaMP based on the average image by using an algorithm for detecting circular shapes in images. We then extracted fluorescent time series from the central part of identified neurons (49 pixels). We identified 79,176 neurons across the brain in the experiment described in Figure 5. We calculated the baseline fluorescence trace for each extracted fluorescence trace by taking the rolling percentile of the bottom 30% with a window size of 2 min and then divided the original fluorescent time series by this baseline trace to obtain ΔF/F time series for each neuron.

For the analyses shown in Figures 5B; Supplementary Figure S5B, we focused on neurons whose task-dependent activity modulation was consistent across multiple sessions. For extracting such neurons, we created a matrix of the average ΔF/F in each task period (12 periods) for each session and performed one-way ANOVA across trials for individual neurons. In this way, we identified 36,818 neurons with significant *p*-values (*p* < 0.001) for consistent task-dependent activity modulation across sessions. We then extracted neurons for each brain area by their spatial locations. We classified neurons into three groups in each area by applying a k-means clustering method (n = 3) to the trial average activities of neurons. We then picked a neuron that shows the largest response amplitude from each identified cluster in each brain area and plotted their time series in Figure 5B.

For the analysis shown in Figure 5C, we used the same set of 36,818 neurons identified in the above statistical test for response reliability. We normalized the ΔF/F trace of each neuron by its 99-percentile value and clipped values more than 1. We then applied non-negative matrix factorization to the activity of neurons (n = 4 components). We extracted neurons whose weight is more than 0.2 for each component and color-plotted their locations in the top projection and side projection images of the brain.

For the analysis shown in Supplementary Figure S5B, we used a volumetric stack of red fluorescence in the same fish acquired after the whole-brain imaging experiments described in Figure 5. We extracted 55 RFP-positive neurons among the above 36,818 neurons by using a hand-drawn mask over the area of the dorsal raphe nucleus. We identified the RFP-positive neurons by detecting the red fluorescence by a fixed threshold for mean pixel values (120). We calculated an average ΔF/F trace for different tasks (7-s training, 15-s training, and 30-s training) across sessions for each neuron and further averaged it across them.

## Results

### Microscope Design and the Architecture of PyZebrascope

We developed PyZebrascope for our custom-designed light-sheet microscope (Figures 1C; Supplementary Figure S1, Supplementary Table S1 for parts lists), for which many components are common to those in the previous studies (Panier et al., 2013; Vladimirov et al., 2014; Mancienne, 2021; Markov et al., 2021). Our system is equipped with two lasers, providing light sources for two excitation arms that are required to scan fish brains from the lateral and front sides (Figure 1B). We control the lasers’ on/off by digital input into the lasers. The brightnesses of laser outputs are controlled by 1) setting the power level of the laser using serial communication and 2) diminishing the laser output with different levels of neutral density (ND) filters. These two levels of brightness adjustment allow us to maintain laser output levels in a range causes less power fluctuation. Typically, the excitation beam for the front scanning (Figure 1B) requires less output power due to its narrow scanning range. Each brightness-adjusted beam is then scanned by sets of 2-axis galvanometers for volumetric scanning. The beams expand through a pair of a telecentric scan lens (SL) and a tube lens (TL) and then focus onto the sample through an excitation objective (EO). Fluorescence from the fish brain is collected by a detection objective (DO) placed above the brain, and this detection objective moves along the axial direction in sync with the excitation beam by a piezoelectric drive during volumetric scanning. The fluorescent image is focused onto scientific CMOS (sCMOS) camera through a tube lens (TL) and an emission filter (EF, Figure 1C). We can also add another sCMOS camera in the detection path for multicolor imaging, as shown in our CAD model (Supplementary Figure S1, CAD model files available on request).

**FIGURE 2:**
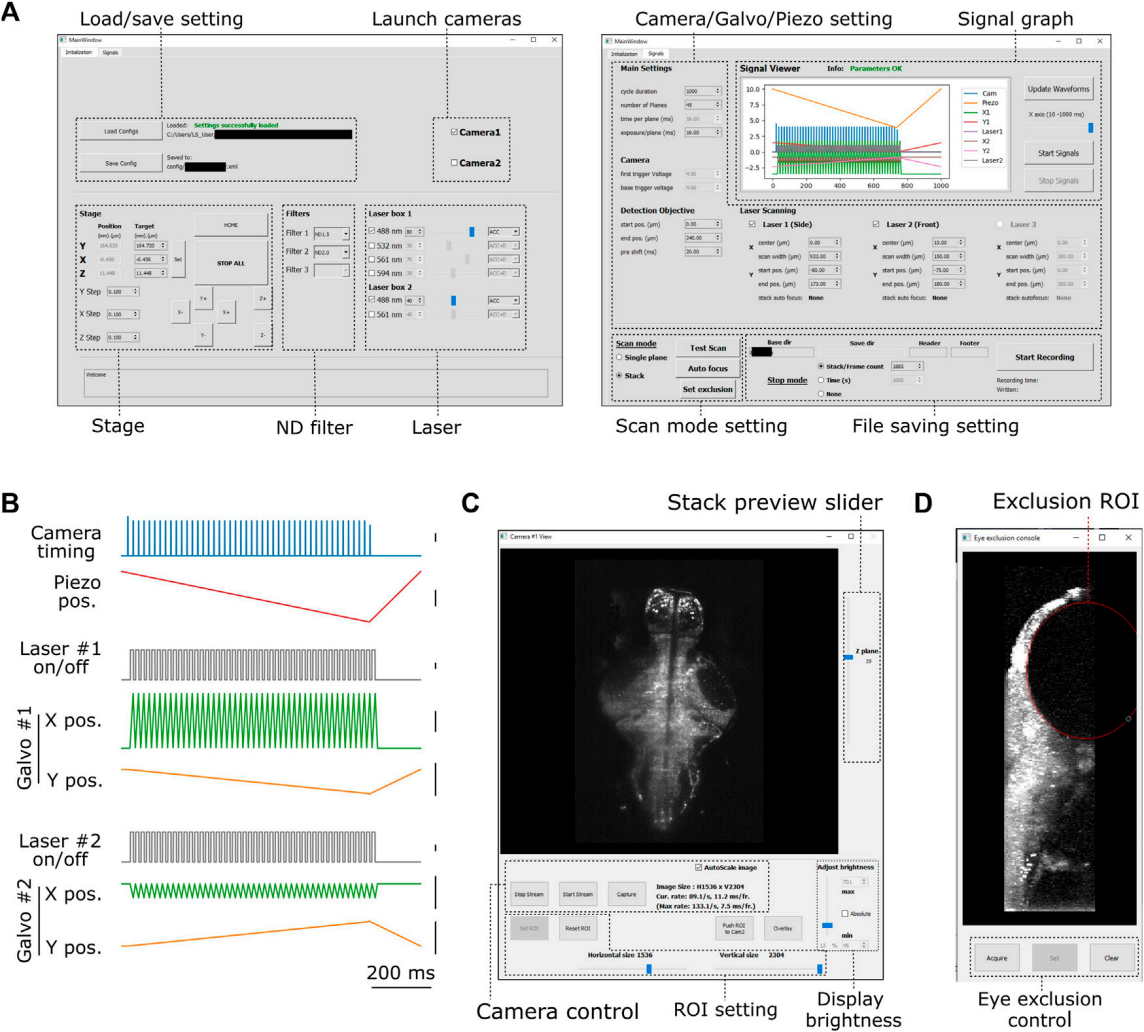
Graphical user interface of PyZebrascope. **(A)** Two main tabbed windows of the user interface of PyZebrascope. The first window provides saving/loading microscope configurations, sample stage control, selection of neutral density filter, and laser control. The second window provides camera exposure control, motion control for the detection objective, scanning control for excitation beams, switch between volumetric and single-plane scans, and FIle saving settings. **(B)** Examples of analog and digital outputs for microscope components during a volumetric scan. Black scale bars next to waveforms represent 1 V. Camera trigger signals have different amplitudes at the start (4.5 V) and the end of a volumetric scan (3.5 V) compared to those in the middle (4.0 V). These varying amplitudes of trigger signals above the camera’s threshold would allow the behavior control software to receive camera trigger signals in parallel and detect the onset of each volumetric scan for synchronization. **(C)** Camera view window. It allows users to view ambient and fluorescent images, scroll through different Z-planes of a volumetric stack, zoom in/out, adjust the image display brightness, set region of interest (ROI) to the camera, and overlay two images in different color channels from two cameras during multicolor imaging. This window can also be stretched to any preferred size. **(D)** Eye exclusion window. The lateral view of a volumetric stack allows the user to set an elliptic exclusion area (red) which will turn off the side laser when it scans over the eye.

PyZebrascope controls the above-mentioned multiple devices through its modular architecture and organized user interface (Figures 1D, 2). It does not require compiling and is launchable from any Python development environment that allows PyQT applications. It controls devices through serial communications (laser setting, filter wheels, sample stage), analog output (galvanometer, camera timing), digital output (laser on/off), and camera interface cables (Supplementary Figure S1). The camera unit (camera.py) controls camera settings through a Python binding (pymmcore, https://github.com/micro-manager/pymmcore) for low-level device interface of μManager (Edelstein et al., 2014). It also acquires images from the camera and saves them in HDF files. The analog/digital output unit (signal.py) generates waveforms and outputs them through a Python binding (ni-daqmx, https://nidaqmx-python.readthedocs.io/) for data acquisition interface devices. Its outputs can be modified to drive alternative components of light-sheet microscopes that operate according to voltage inputs, such as electrically tunable lens (Favre-Bulle et al., 2018) and scanners based on microelectromechanical systems (MEMS) (Migault et al., 2018). Other modules for controlling devices via serial communication (laser.py, nd_filter.py, piezo.py) are written in a generalizable manner so that external users can adapt them to their preferred devices.

The configuration unit (setting.py) allows the users to save all the microscope configuration parameters in an XML file. Savable parameters include the state of all connected devices, the position of the 3-axis stage, the choice of lasers and their output intensity, type of neutral density filters, the region of interest on the camera’s field of view, and parameters for volumetric scanning. This file is also automatically saved for every experiment with time logs. The experimenter can load this setting file later to connect necessary devices and configure all the device parameters automatically. Although the user still needs to fine-tune sample positions and microscope scanning parameters for each experiment, this automatic loading minimizes the users’ efforts in conducting experiments consistently across sessions.

### Sample Preparation and Stage Control

The fish for imaging resides Sample Stage Control a water chamber with glass windows (Figure 1C). Within the water chamber, the fish is embedded in low-melting agarose on a small pedestal, and this pedestal allows us to expose the fish head after removing surrounding agarose and at the same time record electrical signals from the tail for fictive swim recording (Ahrens et al., 2012; Vladimirov et al., 2014; Kawashima et al., 2016). The user mounts the fish into the water chamber outside the microscope at the beginning of each experiment. Then the chamber is placed on a motorized 3-axis stage, which brings the fish to the focus of the detection and excitation objectives (Supplementary Figure S1).

The PyZebrascope sample stage unit (stage.py) moves the motorized stage holding the water chamber according to the user’s commands from the software interface. It also allows users to automatically move the stage between stereotypical positions, such as a home position for replacing water chambers and an imaging position for performing experiments. During imaging experiments, the water chamber, the detection objective, and the excitation objectives need to be placed in close physical proximity. Therefore, the stage unit moves the stage in a constrained manner to avoid the risk of accidental collisions between these components.

### User Interface and Waveform Outputs of Pyzebrascope

We designed graphical user interfaces based on QT Designer (Figure 2, https://doc.qt.io/qt-5/qtdesigner-manual.html). All of the above device parameters are organized in two tabs in the main window (Figure 2A). Waveforms of analog and digital outputs (Figure 2B) can be viewed in the user interface window. We implemented a waveform generator module (signal.py) that synchronizes the beam scanning to the full opening of the rolling shutter in the sCMOS camera. It also moves the piezoelectric drives and the beam scanning in the axial direction in a continuous manner (Figure 2B) rather than in a step-wise manner because the piezoelectric drive cannot move the weight of the detection objective with sub-millisecond accuracy (Supplementary Figure S3). Its continuous motion without frequent accelerating/decelerating events provides a better match of axial positions between the detection objective and beam scanning. Also, the waveform for moving piezoelectric drive can be shifted by a user-defined time (10–20 milliseconds) compared to those for scanning galvanometers to compensate for the movement delay between the movement of the piezoelectric drive and its analog input (Supplementary Figure S3). This module uses Numpy (Harris et al., 2020), a common library for array programming for researchers, to generate waveforms and can be flexibly edited in the code (signal.py) to enable different types of scanning patterns such as bidirectional scanning for faster volumetric imaging (Saska et al., 2021).

The camera view window (Figure 2C, camview.py) will pop up separately when the camera is turned on. It allows the users to view the ambient and fluorescent images of the sample and adjust the display brightness and zoom. It also enables the inspection of the quality of the volumetric stack by using a slider for changing the displayed Z planes. PyZebrascope supports two cameras in the detection path for multicolor imaging, and this camera view window can display images from two cameras separately or overlay them in RGB color channels.

PyZebrascope also has a dedicated module and interface for eye damage prevention (eye_exclusion.py) (Figure 2D). This feature is necessary for the side laser whose scanning pattern overlaps the eye during whole-brain imaging (Figure 1B) and not for the front laser that only needs to scan a narrow width between the eyes. In this interface, the experimenter looks at the side projection of a volumetric stack and draws an elliptic region of interest (ROI) to set where the lateral laser should be turned off (Figure 1B). This module automatically calculates the timing for turning off the laser across multiple Z-planes based on the set ROI. Thus, this interface prevents laser illumination into the eye while performing experiments, enabling behavioral tasks that depends on visual features presented to the fish.

### Acquisition, Buffering and File Saving of Imaging Data

One of the challenges in neural activity imaging using light-sheet microscopy is its high image data throughput. Modern scientific CMOS cameras for light-sheet microscopy can acquire several megapixels per frame at the speed of over a hundred frames per second, which amounts to a data throughput of several hundred megabytes per second (MB/s). This high load poses significant challenges in ensuring continuous data flow from camera acquisition to file writing while avoiding writing delays and memory overflows.

**FIGURE 3:**
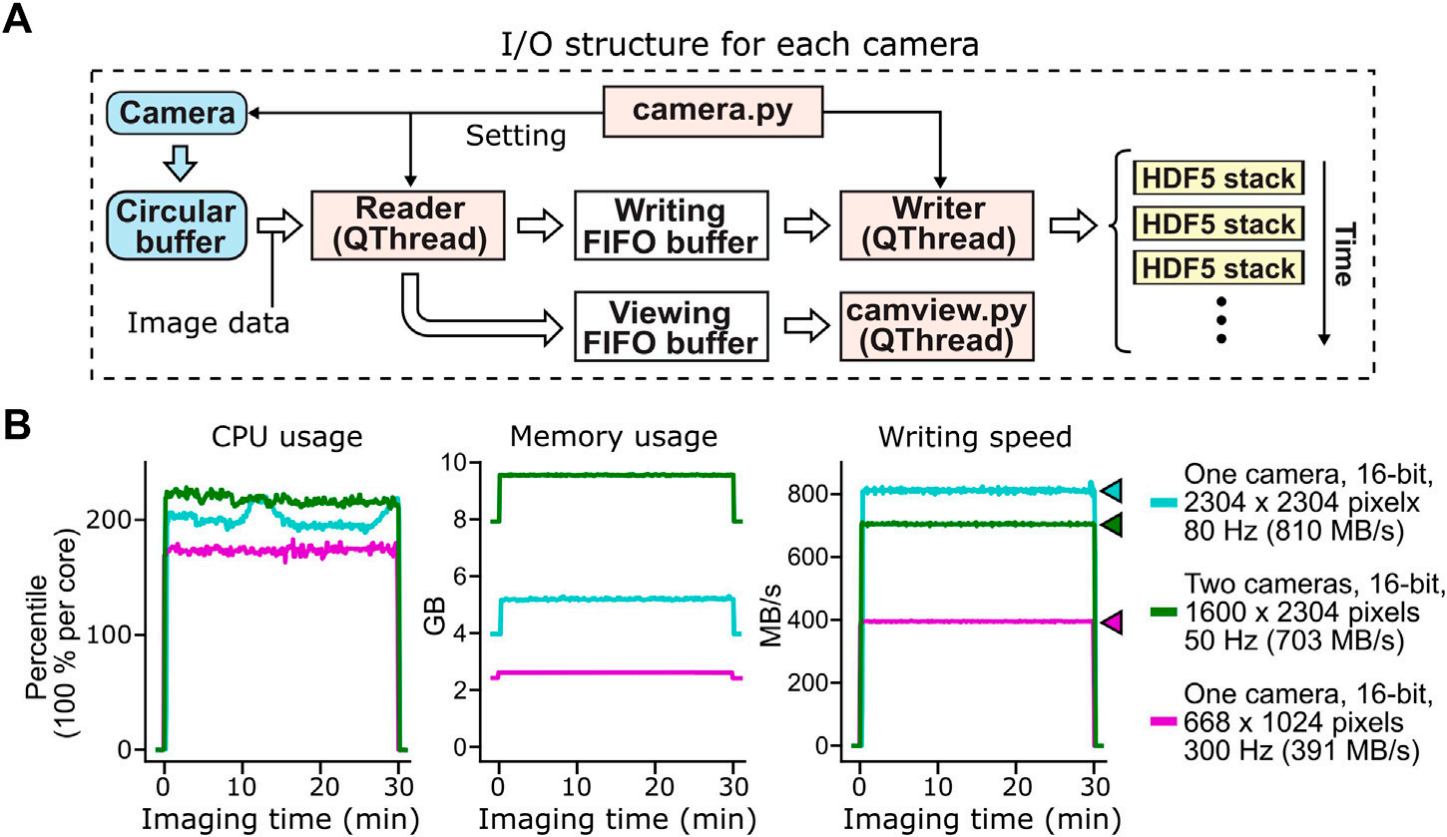
Acquisition, buffering and file saving of imaging data. **(A)** The flow of image data acquisition, buffering and file saving. PyZebrascope uses three threads and three memory buffers to handle imaging data for each camera. Image data first move from the camera to the camera driver’s circular buffer. Once the image arrives at the circular buffer, Reader thread immediately transfers it to the first-in-first-out (FIFO) buffer in the system memory for file writing. Reader thread also intermittently copies the same image data to another FIFO buffer for previewing on the software interface. Writer thread takes the image data from the writing FIFO buffer and saves them into HDF5 files. Each HDF5 files stores a set of images from each volumetric scan or a set of 100 images for single-plane imaging. **(B)** Stable CPU usage, system memory usage, and data writing by PyZebrascope during high data throughput. We tested three conditions that induce higher loads than typical whole-brain imaging experiments demonstrated in Figure 5: full-frame 80 Hz imaging from one camera (cyan), simultaneous 50 Hz imaging from two cameras (green), and 300 Hz imaging from one camera. We measured CPU usage (left), system memory usage (center) and disk writing speed (right) before, during, and after a 30-min recording session for each condition. Colored arrows next to the graph of disk writing speed represent data rates from the camera for different conditions.

PyZebrascope handles such flow of imaging data using three threads and three buffers for each camera (Figure 3A). The reading thread continuously transfers acquired images in the circular buffer of the camera to a first-in-first-out (FIFO) buffer in the system memory for file writing. This system buffer based on the Python queue module is advantageous compared to the camera’s circular buffer in that it allows access from multiple threads, does not require buffer preallocation, and does not allow overwriting of previously queued data. The writing thread continuously takes the image data from this FIFO system buffer and saves them into Hierarchical Data Format 5 (HDF5) files in uncompressed form. We chose this file format because writing in HDF5 format using h5py library (https://www.h5py.org/) yielded higher data throughput than writing in other formats, such Tagged Image File Format (TIFF), at the time of our prototyping. Each HDF5 file stores a set of images for each volumetric scan or a set of 100 images for single-plane imaging. In parallel with these file writing processes, the reading thread intermittently copies image data to another FIFO buffer on system memory (Figure 3A). This buffer provides image data to the camera view thread for displaying acquired images on the software interface (Figure 2C) during imaging experiments.

This structure for handling image data, when combined with a high-speed solid-state drive (>3 GB/s writing speed, see Methods for our computer specifications), achieved a throughput of over 800 MB/s during the continuous acquisition of full-frame (2304 by 2304 pixels), 16-bit images at the speed of 80 frames per second (Figure 3B). PyZebrascope’s CPU and memory usage remained stable during a 30-min recording, and its disk writing speed matched the data rate from the camera throughout the recording. We also achieved stable data throughput for multi-camera imaging (1,600 by 2304 pixels, 16-bit, 50 frames per second) and high-speed imaging (668 by 1,024 pixels, 16-bit, 300 frames per second). We expect it will be possible to scale up image dimensions or frame rates further by, for example, implementing a camera acquisition mode with a lower bit depth or by optimizing file saving algorithms.

### Synchronization With Behavioral Recording

We synchronize behavioral control software to the image acquisition of PyZebrascope by diverging the camera trigger cable to an analog input channel for the behavioral software (Supplementary Figure S4A). The waveform generator (Figure 2B) of PyZebrascope assigns varying voltage amplitudes to camera trigger pulses above the triggering threshold (3.3 V). This varying pulse amplitude allows the behavioral software and post-processing algorithms to detect camera timings for the first axial plane in a volumetric scan (4.5 V), those for the second to the last axial planes (4.0 V), and those that terminate the acquisition of the last axial plane (3.5 V) (Supplementary Figure S4B). For example, behavioral software can switch the behavioral task by counting the number of volumetric stacks, rather than the number of all the pulses, by detecting 4.5 V pulses at the start of the acquisition based on voltage thresholding.

The waveform generator module (signal.py) allows the addition of digital outputs necessary for synchronizing image acquisition to other types of behavioral software. It is possible to add separate digital outputs for the start of the volumetric scan or individual image acquisitions as described above by adding binary arrays to the digital output channels.

### Automatic Alignment of Excitation Beams to Image Focal Planes

**FIGURE 4:**
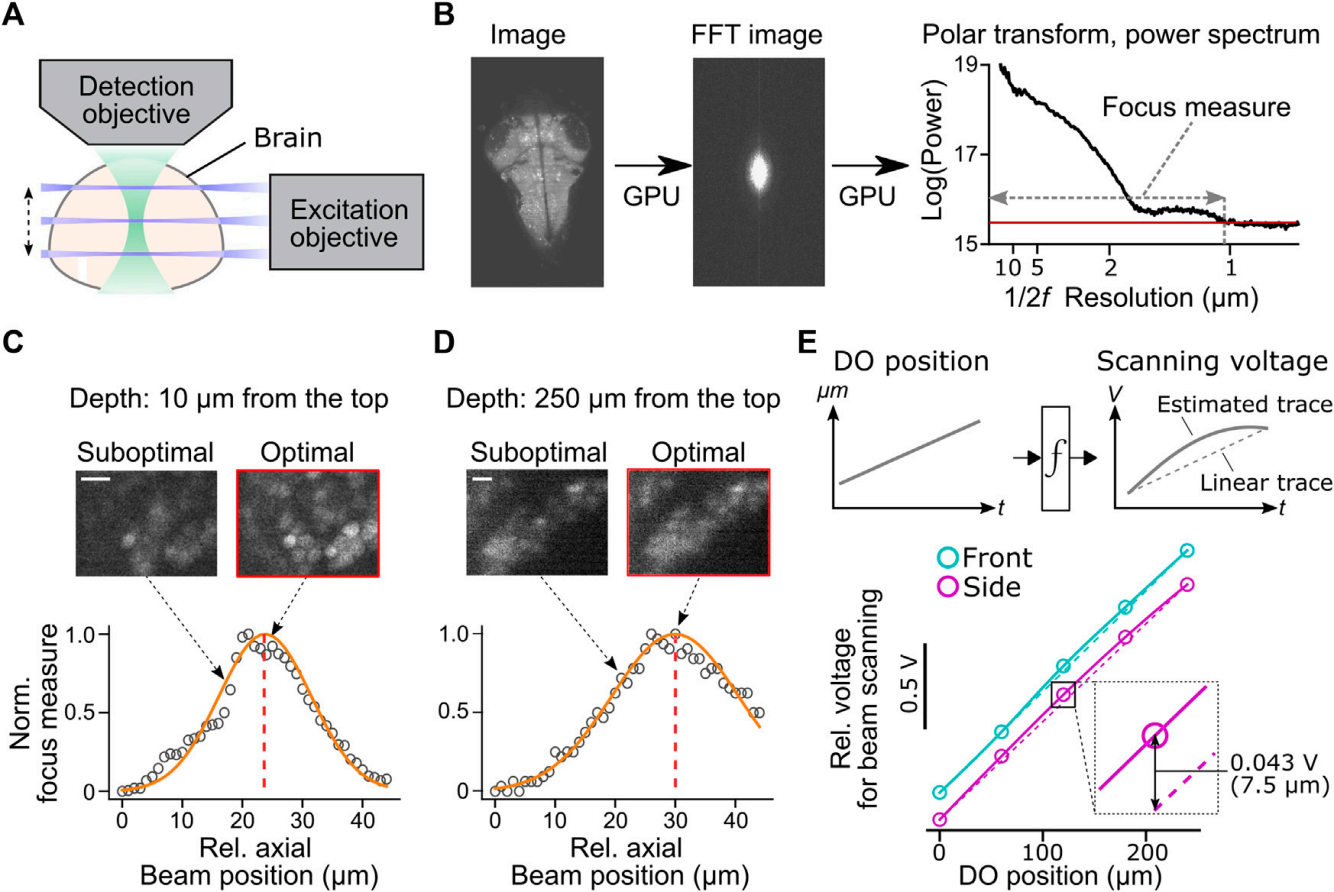
Automatic alignment of excitation beams to the image focal plane. **(A)** Schematic of the alignment between the excitation beams and the detection objective based on acquired fluorescent images in the brain. **(B)** Calculation of focus measures. We apply a Fourier transform to the image, followed by a polar transform to the resulting power spectrum and a 1D projection along the angular axis. Then we calculate the width of the power spectrum above the threshold as a focus measure. Images with the finest resolution yield a higher focus measure. **(C)** Detection of the optimal beam position for the dorsal part of the brain. Normalized focus measures at different beam positions (circles), Gaussian fit (orange line), and the best focus (red dashed line) are plotted. Sample images from different beam positions are shown on the top. Scale bar, 10 μm. **(D)** Same plots as **(C)** for the ventral part of the brain. Scale bar, 10 μm. **(E)** Top, estimation of the nonlinear function between the position of the detection objective (DO) and analog output voltage for the axial scanning of the excitation beams during a volumetric scan. The estimated function (solid lines) may show nonlinearity compared to linear estimation based on the start and end position of the detection objective (dotted lines). *Bottom*, we estimated nonlinear functions between the positions of the detection objective (DO) and analog output voltage for axial beam scanning in the brain of a zebrafish. We determined optimal voltage output at five positions of the detection objective during a volumetric scan of the front beam (cyan) and side beam (magenta) independently and estimated transformation functions by cubic interpolation. The resulting functions (solid lines) showed nonlinearity compared to linear estimations (dashed lines). *Bottom inset*, the difference between the optimal analog output and the linear estimation was as large as 0.043V, which amounts to 7.5 μm of the axial position of the side beam, at the middle plane of a volumetric scan.

The Python software ecosystem offers a variety of highly optimized libraries for image processing and machine learning. Such distinct advantages of using Python, as well as the modular architecture of PyZebrascope, enable the implementation of advanced algorithms for microscope control and online image analyses. As a proof of concept to demonstrate this advantage, we developed an autofocusing module (auto_focusing.py) that adjusts the axial position of the excitation beam in the sample to the best focus of the detection objective (Figure 4A). This procedure is usually a time-consuming manual step during the preparation of whole-brain volumetric imaging. The experimenter needs to align the position of the excitation beam from the superficial part to the deep part of the brain. This alignment is necessary for both the front and side excitation beams. In addition, images at the bottom part of the brain are typically blurry due to the diffraction of fluorescence through the sample, which makes the alignment indecisive for the experimenter.

We implemented a novel automatic algorithm to assist such a time-consuming alignment process by using an image resolution measure based on Fourier transformation (Mizutani et al., 2016) (Figure 4B). Optimal beam alignment yields a higher resolution of fluorescent images, which results in higher powers in the high-frequency domain in images. This algorithm first Fourier-transforms the fluorescent image to obtain its 2D power spectrum. A polar transform is applied to the 2D power spectrum and is projected onto 1D by averaging along the angular dimension. We then use the logarithm of the obtained 1D projected array to quantify image resolution. The threshold for quantifying image resolution is defined as the sum of the mean and three times the standard deviation of the baseline part of the power spectrum. We then count the number of points above the set threshold and use this number as an image resolution measure (Figure 4B). We implemented this algorithm using a library for GPU computing (CuPy, https://cupy.dev/) to minimize computation time for Fourier transform and polar transform.

Our auto-focusing module searches for the best focus by acquiring images at different axial beam positions (41 planes at an interval of 1 μm) for a given position of the detection objective (DO). It calculates the above resolution measure for each acquired plane and normalizes resolution measures to between 0 (poor resolution) and 1 (best resolution) across different planes. The peak position is detected by fitting a Gaussian distribution function to the normalized resolution measures. The center of the estimated distribution was designated as the best focal plane. These sampling and computing processes take less than 2 s in total and accurately detect the best focus for the dorsal (Figure 4C) and ventral (Figure 4D) part of the fish brain. We further applied this technique to estimate the nonlinear function between the position of the detection objective (DO) and voltage commands for axial positioning of the excitation beams during a volumetric scan (Figure 4E). Such nonlinearity occurs because the angle of the scanning galvanometer is not necessarily linear to the axial position of the excitation beam. Our auto-focusing module automatically finds the best axial positions of the excitation beams, independently for the side and front beams, for five different DO positions along the *Z*-axis of a volumetric scan. It then estimates the optimal transformation function between the DO movements and scanning voltage for the excitation beams. Transformation functions obtained in a real zebrafish brain showed nonlinearity (Figure 4E), and the optimal beam position at the middle of a volumetric scan differed from the linear estimation by 7.5 μm, a large enough distance to affect image resolution significantly (Figures 4C,D). This estimated nonlinear function is passed to the waveform generator module (signal.py) as *interp1d* function of Scipy package (Virtanen, 2020) to transform the analog output waveform. Thus, this algorithm allows us to obtain accurate alignment between the objective and the excitation beam beyond manual, linear adjustments based on the start and end position of the volumetric acquisition.

### Whole-Brain Imaging in Behaving Zebrafish

**FIGURE 5:**
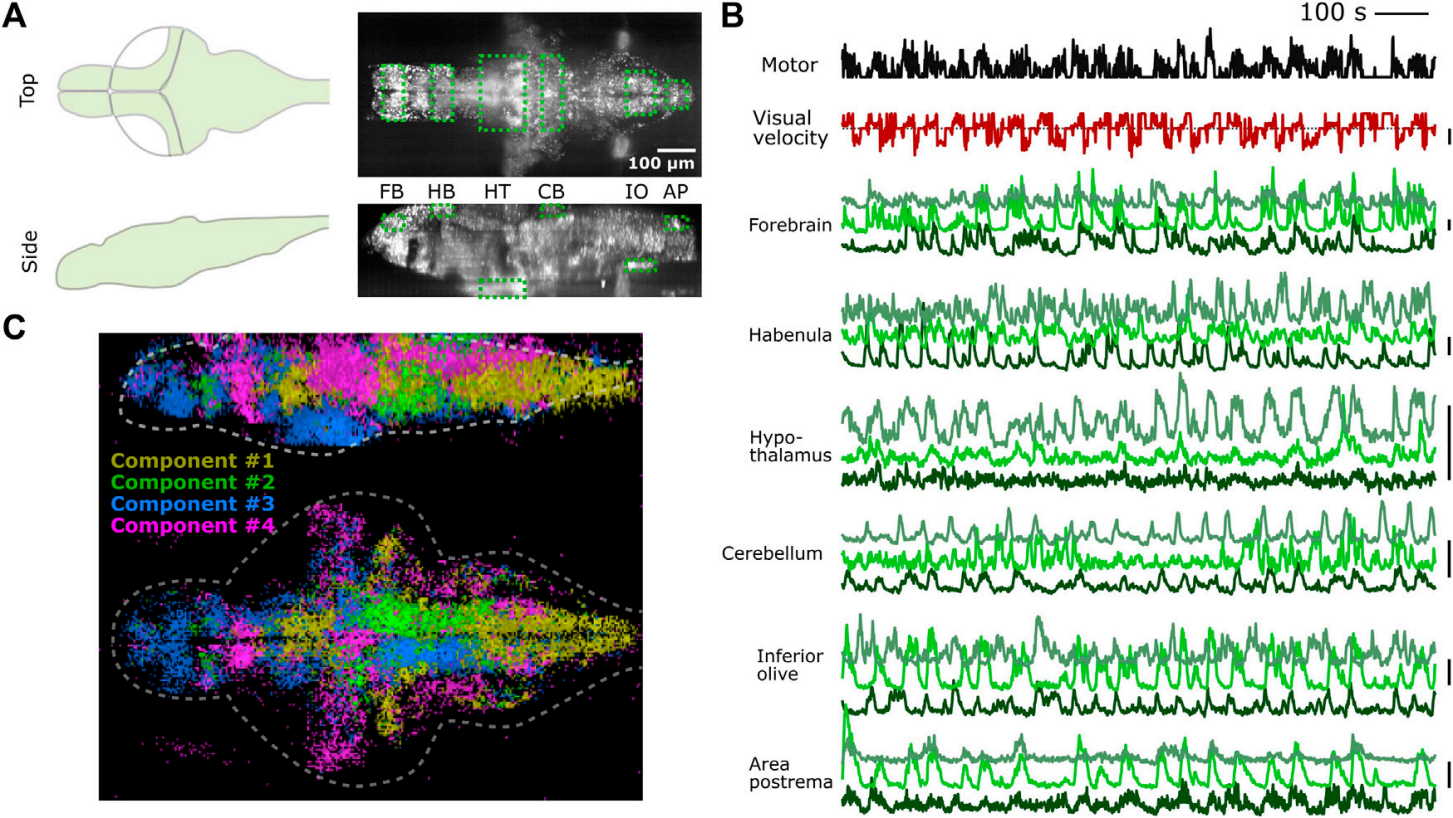
PyZebrascope enables stable recording of neural activity at a brain-wide scale in behaving zebrafish. **(A)** Scanning area of whole-brain imaging. The scan area covers the most dorsal part of the brain (cerebellum = CB, habenula = HB), the most ventral part of the brain (hypothalamus = HT), the most rostral part of the brain (forebrain = FB), and the most caudal part of the brain (Area postrema = AP, inferior olive = IO). **(B)** Simultaneous recording of swimming events (black), the visual velocity of the environment (red), and neural activity traces (green) of representative neurons from brain areas designated in **(A)** during a motor learning task. Three neurons are selected from three groups that show distinct activity patterns from each area based on k-means clustering methods. Scale bars on the right represent 100% change in ΔF/F. Scale bar for visual velocity represents 2 mm/s, and traces below dotted lines represent backward motions of the environment triggered by swimming. **(C)** Whole-brain spatial map of neural activity clusters classified based on non-negative matrix factorization (n = 4) of 36,168 neurons. Detailed descriptions are in the main text and method section.

We tested whether PyZebrascope can perform whole-brain imaging in zebrafish while recording its behavior during a behavioral task (Figure 5A). We used a 6-day old transgenic zebrafish that pan-neuronally express nuclear-localized, genetically-encoded calcium indicators (Dana et al., 2019) (HuC:H2B-GCaMP7f) and co-express red fluorescent proteins in *tph2+* serotonergic neurons (Supplementary Figure S5A). The tested fish was able to react to the visual stimuli presented from below (Figure 5B) and performed the motor learning task described in our previous work (Kawashima et al., 2016). We were able to acquire continuous volumetric scans (45 planes at 1 Hz) across the entire brain (Figure 5A), from the extremities of the dorsal to the ventral regions (cerebellum and hypothalamus, respectively) and from extremities of the rostral to the caudal regions (forebrain and area postrema, respectively).

We analyzed the acquired data by using image registration and segmentation algorithms developed in our previous work (Kawashima et al., 2016). We identified ∼80,000 neurons across the brain, and their neural activity patterns remained stable for the entire duration of the experiment from the above areas of the brain (Figure 5B). These results demonstrate PyZebrascope’s robustness for continuously acquiring large volumetric data over time. We tested the reliability of the data acquired by PyZebrascope by examining whether it is possible to identify behavior-related neural ensembles identified in other studies. We applied non-negative matrix factorization (4 set components) to the neural activity of all neurons across the entire brain and mapped the spatial locations of neurons that have significant weights for the identified components (Figure 5C). We were able to identify a swim-related network in the midbrain and the hindbrain (Orger et al., 2008; Vladimirov et al., 2014) (Component 1), a network in the hindbrain that biases the fish’s swimming to the left side and to the right side (Ahrens et al., 2013; Dunn et al., 2016) (Component 2, 3), and neurons in the optic tectum, the dorsal raphe and the thalamus that respond to visual feedback during motor learning task (Kawashima et al., 2016) (Component 4).

Additionally, we analyzed the activity patterns of *tph2+* serotonergic neurons in the dorsal raphe nucleus that mediate motor learning effect in this behavioral task (Kawashima et al., 2016). We identified 55 RFP+ neurons in the DRN whose task-dependent activity modulation was consistent across multiple sessions (see Methods). Their activity patterns show slow integration during a training period when the fish learns weak swim patterns. Their activity then slowly decay during the delay period of no swimming (Supplementary Figure S5B). These patterns are consistent with the finding of our previous work (Kawashima et al., 2016). These results demonstrate that our open-source PyZebrascope allows us to perform whole-brain neural activity imaging in behaving zebrafish in a quality comparable with the pioneering studies that relied on commercially developed software.

## Discussion

Here we described the development of the open-source Python software, PyZebrascope, that controls a light-sheet microscope designed for neural activity imaging experiments in zebrafish. Its intuitive graphical user interfaces and ease of managing complex device parameters allow the users to minimize efforts in setting up consistent whole-brain imaging experiments across experiments. Its multi-thread structure for handling imaging data achieved a high throughput of over 800 MB/s of camera acquisition and file writing while maintaining stable usage of CPU and memory resources. The choice of Python as a programming language allowed us to implement an advanced microscope control algorithm, such as automatic focusing, which further reduces the tedious efforts for ensuring the quality of multi-beam volumetric scans. These features allowed us to perform whole-brain imaging in behaving zebrafish at a significantly larger scale than the first demonstration of open-source software for light-sheet microscopy in zebrafish (Saska et al., 2021). Its data quality reached at least a comparable level to those demonstrated by commercially developed software (Vladimirov et al., 2014; Kawashima et al., 2016).

PyZebrascope and its components are written in a generalizable manner, enabling research teams that use different types of devices to adapt the code to their configurations. It is available from Github (https://github.com/KawashimaLab/PyZebraScope_public). It only requires pre-installation of Anaconda Python package, μManager package (Edelstein et al., 2014) with a matching version of its Python interface (pymmcore), a Python library for controlling data acquisition board (ni-daqmx), a Python library for GPU computing (CuPy), and a few other Python packages, all free of cost. Therefore, we expect that PyZebrascope will help disseminate the whole-brain imaging technique throughout the zebrafish neuroscience community.

The architecture of PyZebrascope further enables the implementation of advanced microscope control and image processing algorithms. For example, a common issue during whole-brain imaging in zebrafish is the sample’s translational and rotational drift resulting from gravity force, tail motions of unparalyzed fish (Markov et al., 2021), or the pressure of pipette attachment during fictive recording in paralyzed fish (Ahrens et al., 2012; Vladimirov et al., 2014; Kawashima et al., 2016). A small amount of drift, especially in the axial direction along which the volumetric scan is under-sampled, can result in the loss of neurons during imaging because the neuronal diameter in the zebrafish brain is usually less than 5 μm. Our previous work detected such drifts during time-consuming post processing (Kawashima et al., 2016). Given that sample drift usually occurs at a slow rate, it will be possible to monitor it in real-time during the experiment by occasionally sampling the scanned volume. It will also be possible to further compensate for translational drifts by moving the 3-axis stage holding the sample. The effectiveness of such online drift correction is demonstrated in a multi-beam light-sheet microscope (Royer et al., 2015). Therefore, implementing such algorithms likely to increase the throughput and the success rate of whole-brain imaging experiments in zebrafish.

It will also be possible to implement real-time image processing features to identify neurons of specific activity patterns for subsequent neural perturbation experiments at a single-cell resolution (dal Maschio et al., 2017; Vladimirov et al., 2018). Real-time registration and segmentation of individual neural activity across the brain will require large computing resources and may not be feasible on the microscope control computer. Nonetheless, it will be possible to calculate, for example, trial-averaged neural activation maps based on a simple recurrent formula if the behavioral events or sensory stimulus occur at regular volumetric scan intervals. Once we identify neurons that show specific activity patterns, we can further laser-ablate or photo-stimulate these populations using an open-source Python resource for holographic two-photon stimulation (dal Maschio et al., 2017; Pozzi and Mapelli, 2021). Such experiments will allow us to investigate the functional roles of neurons that show specific activity patterns beyond what is obtainable by genetic labeling of neurons.

Lastly, PyZebrascope will enable further development of voltage imaging techniques in zebrafish. Voltage imaging requires high image resolution, high photon collection efficiency, and high imaging speed at the rate of at least 300 frames per second (Cohen and Venkatachalam, 2014; Abdelfattah et al., 2019; Böhm et al., 2022). Light-sheet microscopy has the potential of realizing these conditions at a brain-wide scale in zebrafish. For example, it will be possible to implement a custom module that performs single-plane high-speed imaging in a sequential manner across different depth while the zebrafish perform a simple, stereotypical sensorimotor task as demonstrated for calcium imaging in zebrafish (Ahrens et al., 2012). Implementing such a capability will advance our understanding of how whole-brain neural dynamics control animal behaviors at a millisecond timescale.

## Data Availability

The raw data supporting the conclusions of this article will be made available by the authors, without undue reservation.
