# Supplementary material for: PyZebrascope: An Open-Source Platform for Brain-Wide Neural Activity Imaging in Zebrafish
Source: Front Cell Dev Biol. 2022 May 19;10:875044. doi: 10.3389/fcell.2022.875044 (PMC9161555; doi:10.3389/fcell.2022.875044)
Supplement: Supplementary file 1 [file DataSheet1.pdf]

Supplementary Figure 1: 3D rendering of our custom light-sheet microscope

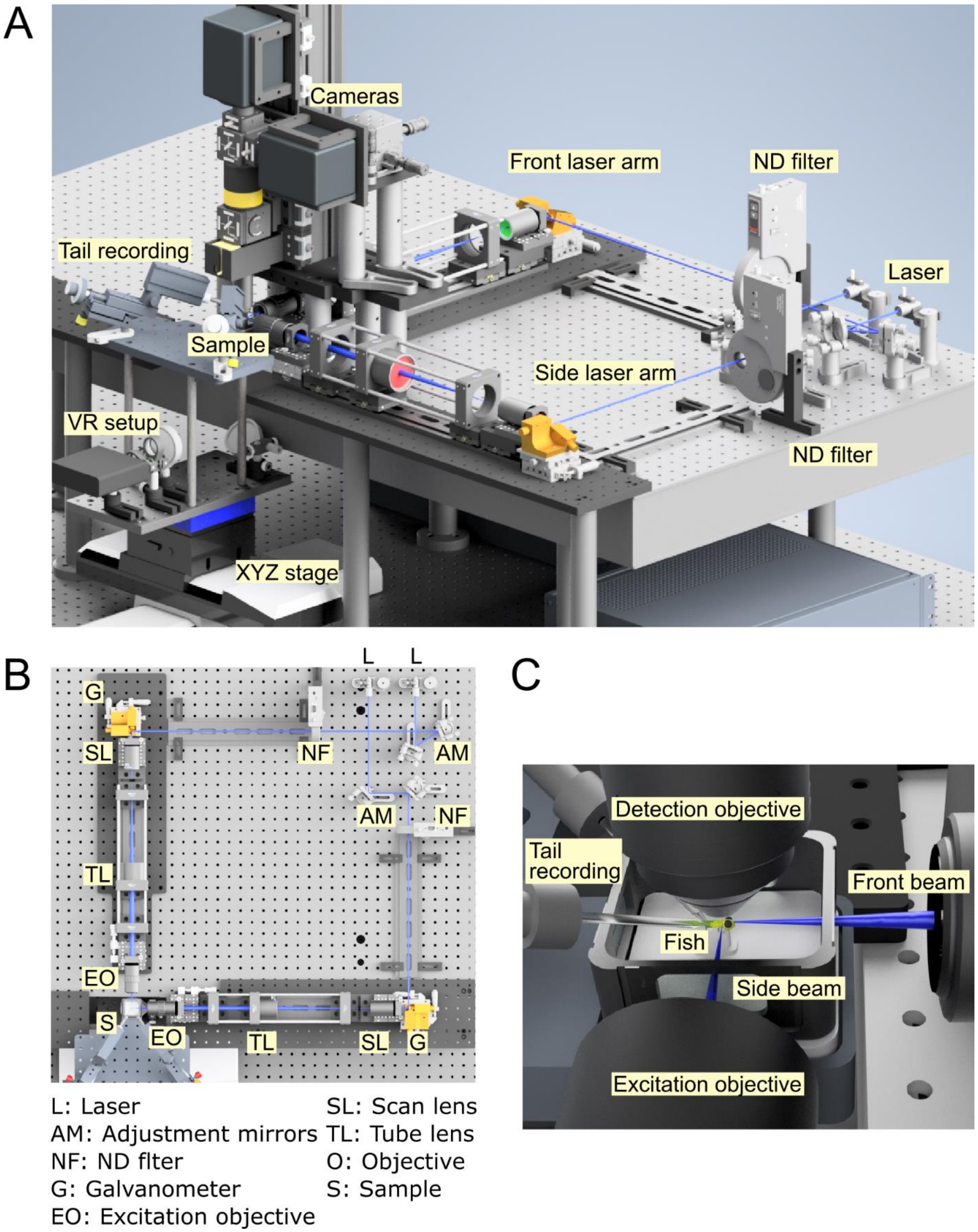

(A) A rendered view of the microscope. **(B)** The optical path of the excitation beams. **(C)** Illumination of two excitation beams into the brain of zebrafish in a water chamber.

Supplementary Figure 2: Wiring diagram of our custom light-sheet microscope.

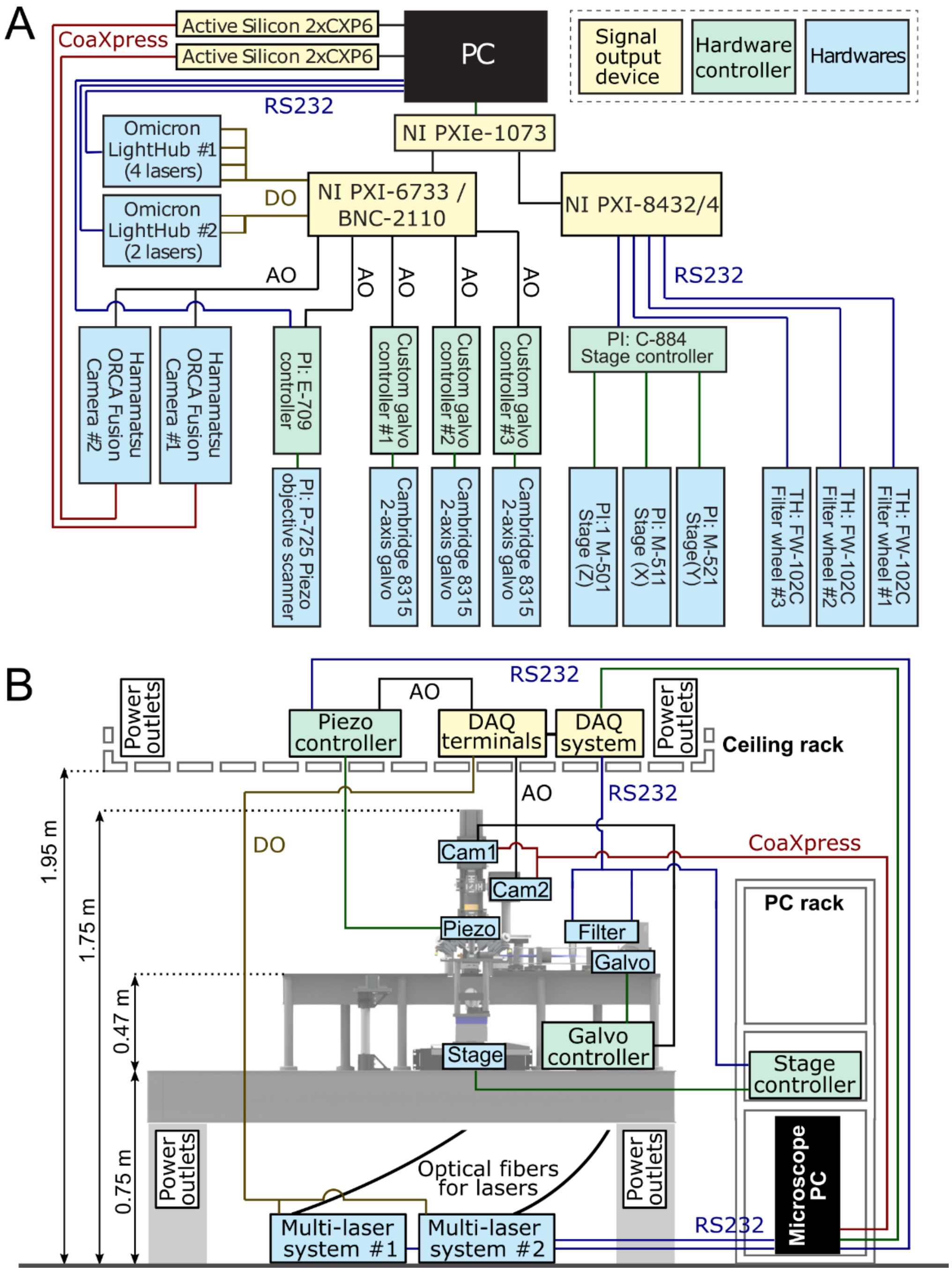

(A) Wiring diagrams for signal input/output devices (yellow), hardware controller (green) and microscope hardware (blue). CoaXPress, camera connection via CXP6 coaxial cables (red line); AO, analog outputs via BNC or SMA coaxial cables (black line); DO, digital outputs (beige line); RS232, serial communication via DB9 or USB cables (blue line). Device-specific cables are drawn in green. **(B)** Physical layout of microscope components on an optical table, the floor, a ceiling rack, and a computer rack. Locations of power outlets are shown as white boxes. Components and cables are drawn in the same color scheme as in (A). Not all the signal and power cables are drawn in this diagram.

Supplementary Figure 3:

The mismatch between step-wise voltage inputs and the actual positions of the piezoelectric scanner

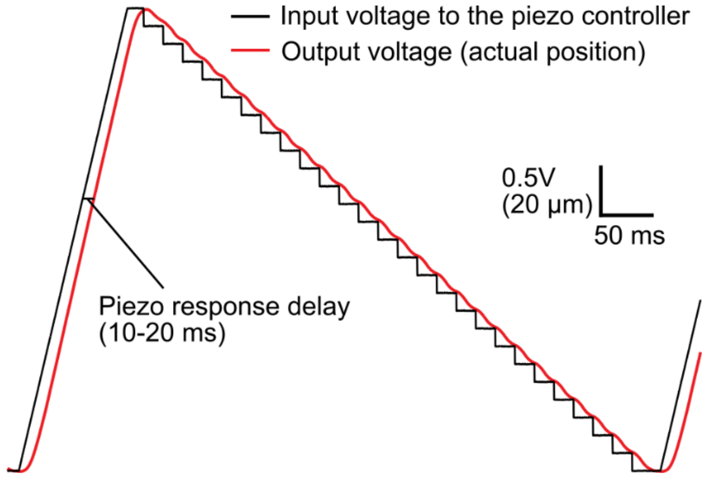

Delayed motion of the piezoelectric scanner for the detection objective. Step-wise analog inputs to the controller (black) during volumetric scanning in the light sheet microscope do not result in precisely matched motion of the piezoelectric drive (red). Its motion is delayed by 10 to 20 milliseconds and is continuous rather than step-wise due to the physical limitation of the piezoelectric drive with a weight load of the objective lens. This limitation of the piezoelectric drive indicates that the continuous smooth motion of the piezoelectric scanner and axial beam scanning shown in Figure 2B, rather than their step-wise motions, yields better matching of the axial position of the detection objective and horizontal excitation beam.

Supplementary Figure 4: Synchronizing behavior software to camera trigger signals

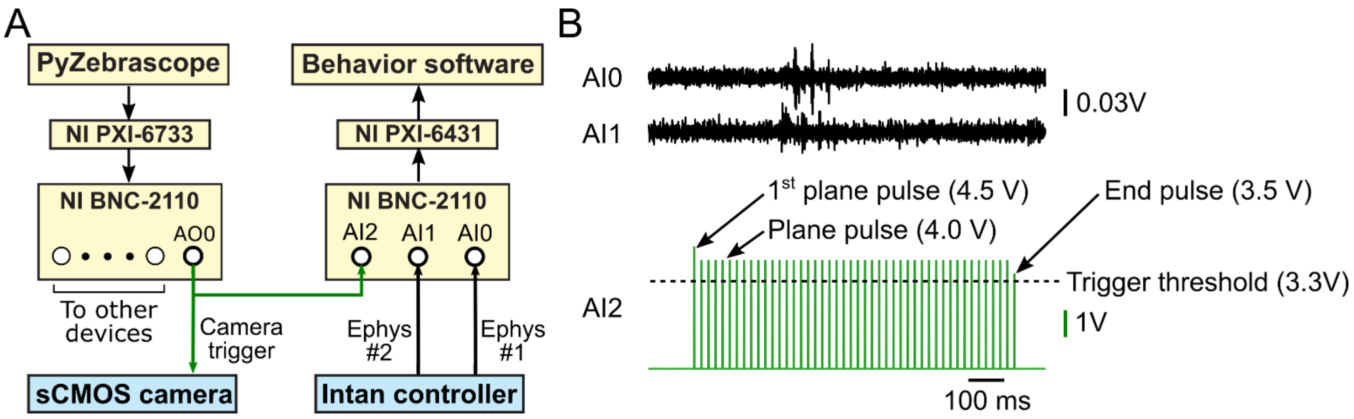

(A) Wiring diagram for synchronizing behavioral software to camera trigger signals. A cable for camera trigger signals (Figure 2B) diverges to an analog input of the data acquisition board for behavioral recording software. **(B)** The behavioral recording software records analog inputs for fictive swim signals (AI0, AI1 for left and right sides, respectively) and camera trigger signals (AI2) at the sampling rate of 6 kilohertz. The camera trigger pulses have varying amplitudes above the threshold for triggering camera acquisition (3.3V). The first pulse for a stack acquisition, which triggers the camera exposure for the first axial plane, has an amplitude of 4.5 V. The second to the last pulse for axial plane acquisition has an amplitude of 4.0 V. The end pulse for finishing the acquisition of the last axial plane has an amplitude of 3.5 V. These varying amplitudes allow users to, for example, synchronize behavioral task events to the start of volumetric scans by using voltage thresholding, while still recording the complete timing information of all camera acquisitions.

Supplementary Figure 5:
Population activity of *tph2*+ serotonergic neurons in the DRN during a motor learning task

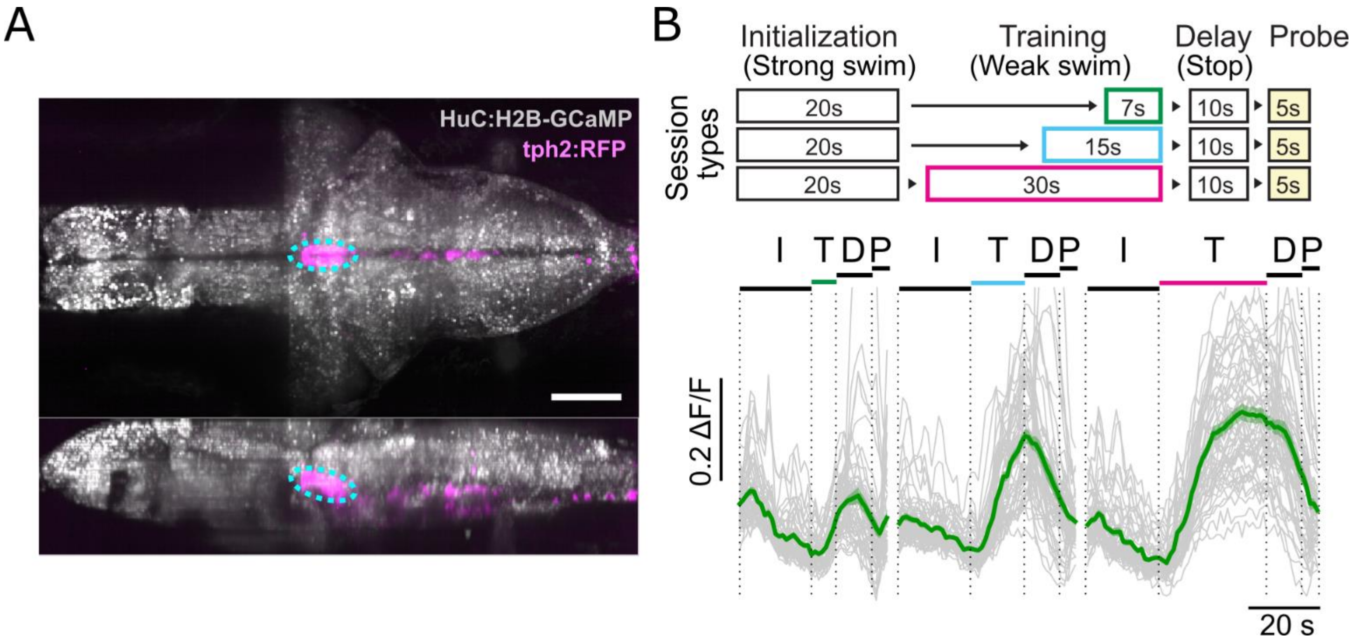

(A) Expression of red fluorescent protein (RFP) in *tph2*+ serotonergic neurons in the whole-brain imaging data presented in Figure 5. **(B)** Trial-averaged activity of 55 *tph2*+ serotonergic neurons during the short-term motor learning task in the whole-brain imaging data in Figure 5. Gray lines represent activity traces for individual neurons. Green lines and shadows represent the average and standard error of the mean across neurons, respectively. These neurons' activity is suppressed during the initialization period (I) when the fish shows a vigorous swim pattern. Their activity slowly integrates during the training period (T) when the fish learns a weak swim pattern. The training duration varies from 7 seconds to 30 seconds across trials. Longer training resulted in higher persistent activity during the delay period (D) and the probe period (P).

Supplementary Table 1: List of major parts for our light-sheet microscope

Item	Details	Supplier
**PC**		
Windows 10 PC	Two Xeon 6244, 192 GB RAM, 15 TB SSD, 10GbEconnection	Access Technology ltd, Israel
**Optical table**		
Custom optical table	Custom RPR table Top 4.0' x 7.0' x 8.0"	(Manufacturer) Newport, USA (Local supplier) New technology, Israel
Vibration isolator	S-2000A-422	
Optical breadboard	Custom breadboard 2.5' x 5.0' x 4.32"	
Optical support rod	XP-10	
**DAQ**		
PXI chassis	NI PXIe-1073	(Manufacturer) National Instruments, USA (Local rep) STEM-Innovation, Israel
Analog inputs for electrophysiology	NI PXIe-6341	
Analog outputs for device control	NI PXI-6733	
Serial inputs/outputs	NI PXI-8432/4	
Connector block	NI BNC-2110	
**Camera**		
sCMOS camera	ORCA Fusion BT	Hamamatsu Photonics Israel ltd, Israel
Frame grabber	Active Silicon 2xCXP6-2PE8	
**Laser**		
Laser combiner #1 (four wavelengths)	LIGHTHUB+ laser engine (488, 532,561 and 594 nm)	Omicron-Laserage Laserprodukte GmbH, Germany
Laser combiner #2 (two wavelengths)	LIGHTHUB+ laser engine (488,561 nm)	
Neutral density filter	NDQ-100, 150, 200, 300, 400	CVI laser optics, USA
Laser goggles	Cat # : DYE, GRY, ZS2	NoIR Laser Company, USA
**Scanning unit**		
2-axis galvanometer	6SD11872 kit (8315K galvanometers with silver-coated 5 mm mirrors, single axis servo drivers)	(Manufacturer) Cambridge Technology, USA (Local supplier) New technology, Israel
Left-handed XY mount	61021505L20	
2-axis manual translation stage	TSD-602S / SD-602SR	OptoSigma Europe SAS, France
Custom mount	Aluminum mount between manual stage and galvanometer mount	Physics Core facility at Weizmann Institute.
Custom linear power supply	Linear 28V power supply for two-axis galvanometer (www.janelia.org/open-science/galvo-driver).	
**Sample Stage**		
3-axis stage	M-521.DD1 (Y) M-511.DD1 (X) M-501.DG1 (Z) C-844.4DC (Controller)	(Manufacturer) Physik Instrumente, Germany (Local supplier) GOA-Tech, Israel
Projector	Optoma LV130	Videoset tech, ltd. Israel

Wratten filter #25 (red)	Cat # 53699	(Manufacturer) Edmund Optics, USA<br>(Local supplier) Prolog Optics, Israel
Custom chamber for zebrafish	Delrin chamber, acrylic fish pedestal, acrylic plate, polycarbonate base	Physics Core facility at Weizmann Institute.
Custom stage for fish chamber	Anodized aluminum	
**Electrophysiology setup**		
3-axis manual manipulator, clamp, headstage mount	MX130R, MX130L, MXB-3h, MXC	(Manufacturer)<br>Siskiyou Corporation, USA<br>(Local supplier)<br>Lahat Technologies, Israel
Electrode holder	Axon HL-U	Molecular Devices, USA
Amplifier and interface board	RHD2000, RHD2132	Intan Technologies, USA
1 mm pins, jacks, sleeves	08F150, 20F2610, 08F162	Bürklin Elektronik, Germany
Custom headstage	3D printed, PLA	
**Excitation and detection optics**		
Breadboard for excitation optics	MB636	(Manufacturer)<br>Thorlabs Inc, USA<br>(Local supplier)<br>Rosh electroptics, Israel
Damped post under galvanometer	DP14A	
ND filter wheel	FW102C	
Tube lens for detection objective	TTL200-A	
Scan lens	SL50-CLS2	
Tube lens	TL200-CLS2	
Excitation objective	MY5X-802	
1-axis stage	LNR25D	
Filter, dichroic	GFP filter (FF03-525/50-25)<br>RFP filter (FF01-607/70-25)<br>Dichroic mirror (FF556-SDi01)	(Manufacturer)<br>Semrock, USA<br>(Local supplier)<br>Lahat Technologies, Israel
Detection objective	CFI75 LWD 16X W	(Manufacturer)<br>Nikon, Japan<br>(Local supplier)<br>Agentek, Israel
Piezoelectric drive for the detection objective	P-725.4CA (400 µm actuator)<br>E-709 Controller	(Manufacturer)<br>Physik Instrumente GmbH, Germany<br>(Local supplier)<br>GOA-Tech Ltd, Israel
**Miscellaneous**		
Stereomicroscope	Microscope (SM745B-V203)<br>Flexible arm (ASC)	AmScope, USA
Custom mount for detection optics	Aluminum	Physics Core facility at Weizmann Institute.
Miscellaneous custom parts		
Custom laser protection panels	Anodized aluminum sheet	
Optomechanical parts	Mirrors, lens, mounts, rails, cages, rods, tubes, screws	(Manufacturer)<br>Thorlabs Inc, USA<br>(Local supplier)<br>Rosh electroptics, Israel
**CAD model**		
The full CAD model is available upon request.		

Supplementary Table 2: Approximate hardware cost for our light-sheet microscope

Custom PC +15 TB drive	17,000
Optical table & breadboard	16,000
Laser system (4 wavelengths)	51,000
Laser system (2 wavelengths)	30,000
Data acquisition system	13,000
3-axis sample Stage & Piezo drive	32,000
sCMOS cameras (QTY 2)	45,000
2-axis galvanometers (2 sets)	11,000
Objective Lens	5,000
Fluorescent filters	3,000
Neutral density filters	2,000
Miscellaneous optical parts	55,000
Custom aluminum parts and panels	Not included
**Total (USD, excluding VAT)**	**280,000**
